# Optimizing bioinformatic workflows to extract clinically usable gene expression data from targeted tumor RNA sequencing panels: comparison with total RNA-seq in cancer samples

**DOI:** 10.1093/bioadv/vbag197

**Published:** 2026-07-13

**Authors:** Xiaokang Pan, Ashley Patton, Yi Seok Chang, Ryan Stevens, Nehad Mohamed, Matthew Hunt, Daniel Chappell, Yan Hu, Cecelia Miller, Weiqiang Zhao, Matthew Avenarius, Dan Jones

**Affiliations:** James Molecular Laboratory at Polaris, The Ohio State University Wexner Medical Center, Columbus, OH 43240, United States; James Molecular Laboratory at Polaris, The Ohio State University Wexner Medical Center, Columbus, OH 43240, United States; James Molecular Laboratory at Polaris, The Ohio State University Wexner Medical Center, Columbus, OH 43240, United States; James Molecular Laboratory at Polaris, The Ohio State University Wexner Medical Center, Columbus, OH 43240, United States; James Molecular Laboratory at Polaris, The Ohio State University Wexner Medical Center, Columbus, OH 43240, United States; James Molecular Laboratory at Polaris, The Ohio State University Wexner Medical Center, Columbus, OH 43240, United States; James Molecular Laboratory at Polaris, The Ohio State University Wexner Medical Center, Columbus, OH 43240, United States; James Molecular Laboratory at Polaris, The Ohio State University Wexner Medical Center, Columbus, OH 43240, United States; Department of Pathology, The Ohio State University Wexner Medical Center, Columbus, OH 43210, United States; James Molecular Laboratory at Polaris, The Ohio State University Wexner Medical Center, Columbus, OH 43240, United States; Department of Pathology, The Ohio State University Wexner Medical Center, Columbus, OH 43210, United States; James Molecular Laboratory at Polaris, The Ohio State University Wexner Medical Center, Columbus, OH 43240, United States; Department of Pathology, The Ohio State University Wexner Medical Center, Columbus, OH 43210, United States; James Molecular Laboratory at Polaris, The Ohio State University Wexner Medical Center, Columbus, OH 43240, United States; Department of Pathology, The Ohio State University Wexner Medical Center, Columbus, OH 43210, United States; James Molecular Laboratory at Polaris, The Ohio State University Wexner Medical Center, Columbus, OH 43240, United States; Department of Pathology, The Ohio State University Wexner Medical Center, Columbus, OH 43210, United States; The Ohio State University Comprehensive Cancer Center, James Cancer Center and Solove Research Institute, Columbus, OH 43210, United States

## Abstract

**Motivation:**

Targeted RNA sequencing (RNA-seq) is widely used to detect gene fusions in tumors but clinical use of expression data from panels in fusion-negative cases has been limited. Differential gene expression (DGE) profiling from these panels has the potential to improve tumor classification.

**Results:**

To facilitate this application, we compared methods for sequence read counting, gene normalization, and supervised and unsupervised clustering methods to optimize them for smaller gene sets. We derived DGE data from ∼200-gene RNA-seq fusion panels. Among five tools for read counting, featureCounts was the most rapid and robust. For DGE with DESeq2, we compared five normalization strategies and showed the five most stably expressed genes over multiple sets provided optimal centralization. The outputs of the optimized pipeline were then assessed by a newly constructed targeted panel that added a limited number of genes assessing cell lineage and tumor grade. Finally, the optimized pipeline was evaluated using mean centroid and principal component analysis and pathway analysis and compared to outputs from full RNA-seq on a common set of challenging tumors. Comparable tumor clustering was observed with RNA-seq and the redesigned targeted gene panel.

**Availability and implementation:**

The data analyzed during the current study are available from the corresponding author on reasonable request.

## 1 Introduction

RNA sequencing (RNA-seq) by next-generation sequencing (NGS) is commonly used to detect diagnostic or therapy-related gene fusions in formalin-fixed paraffin-embedded (FFPE) tumor samples. To achieve robust validation, rapid sign-out and maximal sensitivity through deep coverage, clinical laboratories typically perform fusion detection using limited/targeted gene sets of 50–500 genes rather than assessing the entire transcriptome particularly given the limited material from many tumor biopsies (Curion *et al.* 2020). Methods for targeted enrichment include bait-probe hybridization or anchored PCR amplification (Heyer *et al.* 2019, Capone *et al.* 2022). However, since relevant gene fusions are present in only a minority of tumors, many targeted RNA-seq studies do not advance the diagnostic workup. Combining gene fusion detection with differential gene expression (DGE) analysis can increase the value of such panels. Clinically relevant goals of DGE, which have been clearly demonstrated for whole transcriptome RNA-seq, include distinguishing tumor site of origin (Wei *et al.* 2014), distinguishing lower grade from higher grade soft-tissue tumors (Wang et al. 2016), and particularly the classification of poorly differentiated tumors where the differential commonly includes carcinoma, melanoma, and sarcoma (Nakamura *et al.* 2026).

The first step in RNA-seq-based DGE analysis is read counting where the number of sequence reads that align to each gene segment are quantified. With bait-probe capture and bridge amplification (Illumina) sequencing, NGS read counts have been shown to correlate with expression as determined by microarray or real-time PCR analysis (Fu *et al.* 2021, de Brito *et al.* 2024). Although bioinformatics tools for extracting valid tumor gene expression from whole transcriptome data have been well-established (Corchete *et al.* 2020), methods optimized for small/targeted RNA-seq datasets are currently limited. Furthermore, the accuracy and reproducibility of any given read counting method needs to be established for a fully validated DGE assay to be used clinically.

Given the wide variation in the quality of FFPE samples, tissue fixation artifacts and admixed non-neoplastic cells dilutional effects on differential gene expression can be significant, especially as compared to fresh/frozen tumor tissues (Maza *et al.* 2013, Li *et al.* 2018). Especially in suboptimal FFPE samples, different normalization strategies can have large effects on outputs. Various normalization or centralization methods for DGE in transcriptome RNA-seq have been applied to reduce bias due to variable tumor content, RNA degradation, and method-based variabilities such as variable capture due to local GC content (Love *et al.* 2014, Evans *et al.* 2018, Bushel *et al.* 2020, Zhao *et al.* 2021). Whether these studies are valid or applicable to limited gene RNA-seq panels has not been well-elucidated.

Presentation of DGE data using diagnostically meaningful outputs that accurately represent differences between tumors are critical for clinical utility. For specific tumor differentials (e.g. poorly differentiated carcinoma versus sarcoma), an unknown case can be mapped onto the relevant categories using heatmaps (Wei *et al.* 2014), nearest centroid (Tibshirani *et al.* 2002), or logistic regression (He *et al.* 2023) as forms of supervised clustering. For more complex tumor differentials, displays by principal component analysis (PCA), a linear dimensionality reduction technique that preserves large pairwise distances and variance in the data, may more easily highlight clustering of individual samples without explicitly specifying distinguishing gene sets. Similarly, t-distributed stochastic neighbor embedding (t-SNE) is another data reduction technique that can potentially easily highlight similarity of a new sample to the reference set of different tumors.

In this study, we employed clinically validated ∼200-gene RNA-seq panels optimized for fusion detection in solid tumors to systemically consider optimal methods of read counting and normalization and consider types of data visualization. We compared the outputs of the optimized pipelines for a targeted panel that included a limited number of genes assessing cell lineage and tumor grade with parallel full RNA-seq and showed highly comparable results in a set of challenging tumors.

## 2 Methods

### 2.1 Assay design, sample sets, and sequencing protocol

All tumor samples and the targeted RNA sequencing assays were employed for routine clinical testing at The Ohio State University James Molecular Laboratory. The additional software tools and analyses were included as clinical pipeline optimization as a quality control project; the additional total RNA-seq sequencing to validate expression signatures were performed per an institutional review board-approved protocol with waiver of consent for use of excess diagnosis-coded samples with delinked identifiers.

The targeted gene fusion NGS panels employed in this study included a custom 190-gene (panel 1) and a 230-gene (panel 2) RNA-seq panel clinically validated at the James Molecular Laboratory to detect diagnostically relevant gene fusions in human tumors. The 190-gene design included only a few typical housekeeping genes (*ACTB, MYH9*) with no gene content explicitly designed for DGE. Over 500 clinical cases, the frequency of reportable gene fusions seen with panel 1 varied by diagnosis approaching 20% for soft-tissue tumors versus ∼5% for carcinomas. To improve utility for gene expression, panel 2 incorporated additional stably expressed/housekeeping and lineage-specific genes particularly to aid in separation of poorly differentiated carcinomas and sarcoma. Among 250 clinical cases using panel 2, 222 genes were usually expressed in tumor samples, with the remaining genes only significantly expressed when a particular fusion was present. The frequency of oncogenic fusion detection was similar to panel 1.

For validation of the design of panel 2 using an optimized pipeline, full RNA-seq (total transcriptome except for ribosomal genes) was performed for sets of unequivocal carcinoma and sarcoma (Table S1, available as supplementary data at *Bioinformatics Advances* online), as well as a set of 32 diagnostically challenging malignment tumors (Table S2, available as supplementary data at *Bioinformatics Advances* online). The favored diagnoses following routine histopathology and immunohistochemistry workup are listed in Table S2, available as supplementary data at *Bioinformatics Advances* online.

All NGS protocols utilized total tumor RNA extracted from pathologist-reviewed, macrodissected FFPE tumor tissue using PureLink FFPE RNA Isolation Kit (Invitrogen/ThermoFisher) and employed library preparation with DNA digestion and ribosomal RNA depletion (KAPA RNA HyperPrep Kit or Watchmaker Polaris). For the targeted panels, this was followed by hybridization with probes covering the full exonic regions of all target genes (xGen, IDT, Coralville, IA, USA) designed through the GOAL consortium (Aisner *et al.* 2023). This size of the panel allowed 10–16 samples to be run on the Illumina NovaSeq 6000 SP flow cell, with adequate depth of coverage (∼20–50 000 mean reads per sample per targeted area, with ∼50 000 000 reads per sample).

To assess the suitability of methods across different gene sets, a range of tumors sequenced with both panel 1 and panel 2 were included. For comparative analysis of performance of different read counting methods, 10 soft-tissue tumor samples from panel 2 were used. For the comparison of normalization and clustering methods, 36 tumor samples from panel 2 were used, with a randomly chosen set of 90 unequivocal sarcomas and carcinomas from various sites. A distinct test set of 10 carcinomas and 11 soft-tissue tumors was used as cell-lineage model evaluators for supervised clustering in DGE analysis (Table S1, available as supplementary data at *Bioinformatics Advances* online).

### 2.2 Initial clinical pipeline

For clinical reporting, we employed a custom pipeline for fusion detection (“FindRNAFusion”): paired-end FASTQ files were downloaded to a high-performance HPE Linux server (256 CPU processors, 755 GB RAM memory, and RHEL 9.0 OS) from a mounted Illumina BaseSpace instance. The raw sequence reads of each sample were mapped to Hg19 (Human Genome version 19) to generate alignment BAM files using the splice-aware STAR aligner (Dobin *et al.* 2013). Each BAM file was indexed by Samtools. The BAM files were then used to make fusion calls using Arriba (Uhrig *et al.* 2021). After filtering out artifacts and low-level fusion calls (<10–20 supporting reads), clinically reportable fusions were summarized in a text report file and a pdf file with graphical display. In this pipeline, the number of sequence reads/coverage in each targeted gene and in each targeted region/exon were also computed from the BAM files using each read counting method, as discussed below. These sequence reads were then normalized, using the methods described below. Subsequently, genes with very low reads were filtered out using a published data-based threshold for maximum-based filters (Rau *et al.* 2013) and the remaining genes with normalized read counts were used for DGE analysis using DESeq2 (He *et al.* 2023).

### 2.3 Read count method comparisons

Five read counting methods, Samtools-view (Danecek *et al.* 2021), CuffLinks (Trapnell *et al.* 2012), HTSeq-count (Anders *et al.* 2015), coverageBed (Quinlan and Hall 2010), and featureCounts (Liao *et al.* 2014) were validated. A Perl script was written to run program commands in parallel with a batch of NGS samples. Samtools-view, which was the validated referent method for panel 1, uses a BED file and a BAM file as input and outputs the number of reads in each targeted range (command line: “samtools view -F 0x04 -q 20 -c -@ 8”). The accuracy of this output was validated by comparing the count of mapped reads to manual inspection/calculation of read depth for a set of genes in the Integrative Genomics Viewer (IGV) (Robinson *et al.* 2011) and for a subset of fusion-positive cases with the proportion of fusion tumor cells counted by in situ hybridization (e.g. *ALK1, RET, ROS1*, *NTRK2*). FeatureCounts uses targeted genes in GTF file and BAM file as input (command line: “featureCounts -p -M -O -C -g gene_name —minOverlap 1 —maxMOp 30 -Q 20 –T 8”). HTSeq-count uses the same files as featureCounts as input (command line: “htseq-count -i gene_name —max-reads-in-buffer 50000000 -s no”). CuffDiff, run with option “—total-hits-norm TRUE,” uses the same files as featureCounts as input. coverageBed was run with option “-count” and “-a BED file” and “-b BAM file” as input.

Concordance of different methods was assessed in panel 2 with the fully validated Samtools-view method as the referent. The precision of each method was assessed using the median rank from the coefficient of variation (CV) values of the 222 routinely expressed genes for each sample (Quinlan and Hall 2010) and for each method. The Pearson correlation coefficient (*r*) was computed to assess the count match between the outputs of Samtools-view and the other methods. These correlation coefficients were calculated using statistical functions in Microsoft Excel.

### 2.4 Normalization comparisons

DESeq2, which outputs relative log expression (RLE), is widely used for differential gene expression analysis and was chosen here because it performs well for larger RNA-seq datasets (Rau *et al.* 2013, Li *et al.* 2022). The calculation of size factors was performed through the “estimateSizeFactor” function. RLE normalization using all genes (“AllGenes”) was compared with results using a highly expressed housekeeping gene *ACTB* (“HK”), a panel of five traditionally used housekeeping genes (*ACTB, MYH9, RANBP2, PRKACA*, and *TFG*; “T-HK5”) and the top 10 highly expressed genes as determined by the average number of reads in all samples in a sample set (“Top10H”) or the top 10 most stable genes in all samples in a sample set (“Top10S”).

Five genes (*CREBBP, BRAF, BRD4, ATF1*, and *CREB1*) that were recurrently noted among the top 10 most stable expressed in a range of different samples for both panels 1 and 2 were also identified (I-HK5, Table S3, available as supplementary data at *Bioinformatics Advances* online). These six normalization approaches were compared to no normalization for their effects on statistical and visualized centralization of the data. The CV% value representing the percentage of the standard deviation to the mean per gene across samples was computed using the formula CV% = STDEV.P × 100/AVERAGE. Total RNA-seq data produced from the same RNA samples and library preparation as the targeted panels were analyzed using the core pipelines normalized using the 5 most stable genes from the targeted panel as normalization and subjected to DESeq2 as above. For pathway analysis, QIAGEN Ingenuity Pathway Analysis (IPA) was used (QIAGEN Digital Insights 2026).

### 2.5 Clustering and visualization

For accuracy of classification of tumor origin, a nearest centroid approach was used to evaluate classification accuracy in a test set of carcinoma and sarcoma (Dabney and Storey 2007, Tibshirani *et al.* 2002). Using normalized gene expression data, the sum of the squared difference of each gene in an individual sample and the diagnostic group mean was calculated. For diagnostic accuracy, the sample was then assigned to the class with the smallest distance from each group mean. Python programs using PANDAS and MATPlotLib were developed to display the centroids, statistics, and centroid PCA plots. Genes were displayed in a fixed order across both panels to ensure direct comparability between classes, with each gene’s centroid expression value plotted as a horizontal line extending from zero to its corresponding value. For visualization, RNA-seq expression values were log-transformed and gene-wise centered to stabilize variance and preserve relative expression patterns.

For unsupervised clustering, PCA, t-SNE, and heatmap-clustering were compared. SRplot (Tang *et al.* 2023) was used to perform PCA and heatmap-clustering. t-SNE-Java (Johnson 2023) was implemented to generate t-SNE graphics for clustering and visualization.

## 3 Results

### 3.1 Optimizing read counting

To convert NGS output from the targeted RNA-seq panels into gene expression data, raw sequence reads obtained from each gene segment were enumerated for each sample using five different tools, including the existing clinical assay method Samtools-view as well as cuffdiff, HTSeq-count, coverageBed, and featureCounts. A sample output from one assay run is shown comparing usability, time-to-output, and resource use (Table S4, available as supplementary data at *Bioinformatics Advances* online). Using eight CPU processors, featureCounts took only 2 minutes to complete as compared to 9 minutes for coverageBed. In contrast, HTSeq-count, CuffDiff and Samtools-view produced outputs in 3, 4, and 6 hours, respectively.

**Figure 1:**
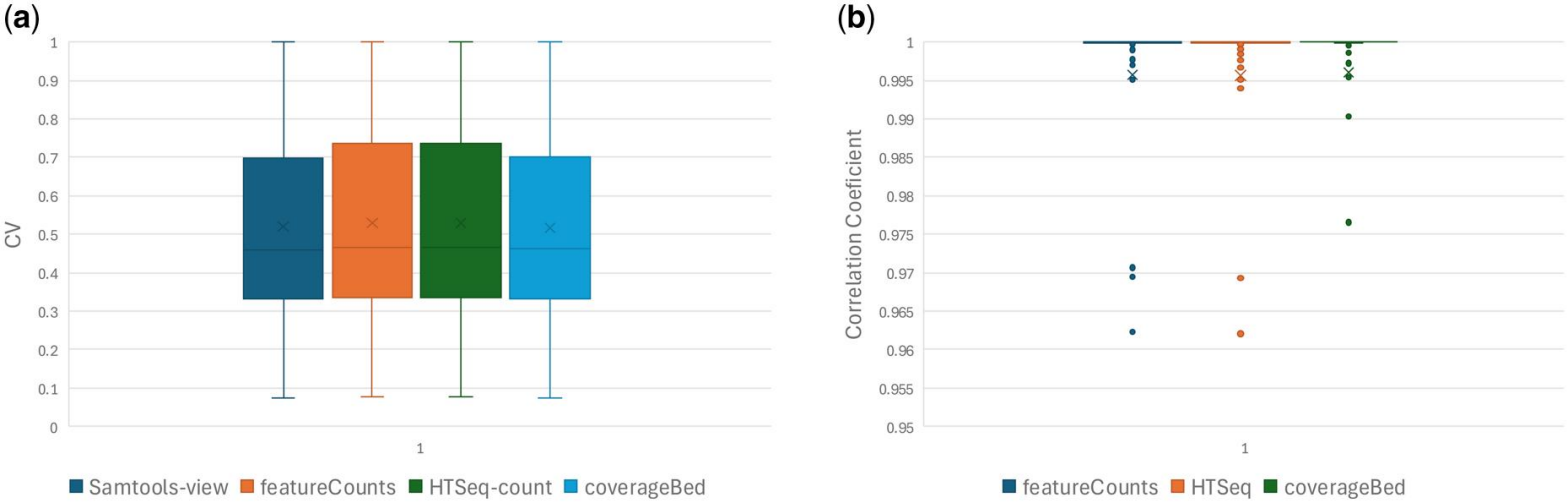
Performance of different sequence read counting methods using a targeted RNAseq panel. (a) Median precision ranks of CV values for the 222 routinely expressed genes in panel 2 are displayed for each read counting tool. (b) Three other tools with acceptable results were compared to the validated reference method Samtools-view for concordance. The ranks of Pearson correlation coefficients for the 222 expressed genes for each method were compared to the referent counting method (i.e. accuracy ranks).

The total reads in the outputs of Samtools-view, featureCounts, HTSeq-count, and coverageBed were similar for 10 samples (Table 1), whereas the read counts from cuffdiff outputs were lower due to some larger genes with high read counts being skipped in the calculation by the software. Excluding cuffdiff, the CV values for each of the 222 expressed genes were computed for each program. The median CV ranks of the 222 routinely expressed genes from the four programs were also nearly identical (Fig. 1a and Table 2). The correlation of each expressed gene in the panel was compared with the validated Samtools-view value using Pearson correlation coefficients and showed high correlations (Fig. 1b); the median rank of the correlation coefficients showed only small differences (Table 2). Considering the performance metrics of output time, precision, and accuracy, featureCounts was rated as the best read counting method for this application and used for subsequent analyses.

**Table 1:** Mapped reads in a run for different read counting methods (panel 2).

Samples	S-107	S-143	S-153	S-175	S-359
**Samtools-view**	23 863 915	84 929 701	38 449 531	103 286 313	61 451 551
**CuffDiff**	3 863 910	3 428 790	62 443 716	7 726 710	4 301 250
**HTSeq-count**	23 505 495	84 600 839	37 801 803	102 395 919	60 991 722
**CoverageBED**	24 306 691	85 794 224	39 043 406	104 359 380	62 160 975
**FeatureCounts**	23 559 590	84 701 209	37 895 291	102 612 244	61 105 419

**Samples**	**S-394**	**S-458**	**S-504**	**S-525**	**S-614**

**Samtools-view**	52 339 994	173 011 118	32 439 398	60 600 048	72 260 163
**CuffDiff**	5 106 000	9 124 866	4 543 470	9 254 736	10 259 952
**HTSeq-count**	51 824 717	171 667 472	31 965 039	59 889 358	71 322 334
**CoverageBED**	53 154 627	174 990 470	32 974 310	61 740 756	73 204 253
**FeatureCounts**	51 932 562	171 937 331	32 037 416	60 076 885	71 446 450

**Table 2:** Precision and accuracy of different read counting methods.^a^

Rank	Counting method	Median rank CV (precision)	Median rank CV (accuracy)	Overall
**1**	Samtools-view	110	na	na
**2**	coverageBed	110	93	203
**3**	featureCounts	110	111	221
**4**	HTSeq	110	113	223

a na, Not applicable as Samtools was the referent method.

### 3.2 Effect of expression normalization strategies on data centralization

Using the raw read counts obtained with featureCounts as input (“NoNorm”), we compared the impact of normalization with *ACTB*, T-HK5, I-HK5, Top10H, Top10S, and AllGenes approaches on RLE data centralization after gene expression analysis with DESeq2.

One readout of effective normalization assessed was a reduction in intragroup variation across tumors of similar types. This was assessed by calculating the mean CV percentage values of read counts before or after normalization for groups of carcinomas, low-grade soft-tissue tumors, and high-grade sarcomas. The lowest CV values for each of the three tumor groups were achieved with the I-HK5, Top10S, and AllGenes normalization methods (Table 3). The intragroup variation for these methods was significantly reduced compared to the NoNorm control condition (*P* < .01, Wilcoxon signed rank test). Intra-tumor group variation was higher than NoNorm for the T-HK5 and *ACTB* normalization methods, with the latter having significantly higher mean CV values for both the carcinoma and sarcoma groups.

**Table 3:** Mean CV percentage values after normalization using DESeq2.^a^

	No norm	ACTB	T-HK5	I-HK5	Top 10H	Top 10S	All genes
**Carcinomas**	93.2	94.0	88.5*	85.0^	**104.7^**	86.7*	85.4^
**Soft-tissue tumors**	82.9	85.3*	84.2	83.4	**89.1^**	82.6	82.2
**Low-grade sarcoma**	100.2	96.7	84.4^	82.5^	101.6	83.3^	81.5^
**High-grade sarcoma**	119.2	**127.9^**	121.7	105.7^	**141.6^**	105.4^	105.8*

a Comparisons by the “estimateSizeFactors” function in DESeq2 using different numbers of genes for normalization. Differences at the 0.01 (^) and 0.05 (*) significance level that were higher (non-bolded) or lower (bolded) than the value without normalization indicated. See Section 2 for normalization abbreviation definitions.

**Figure 2:**
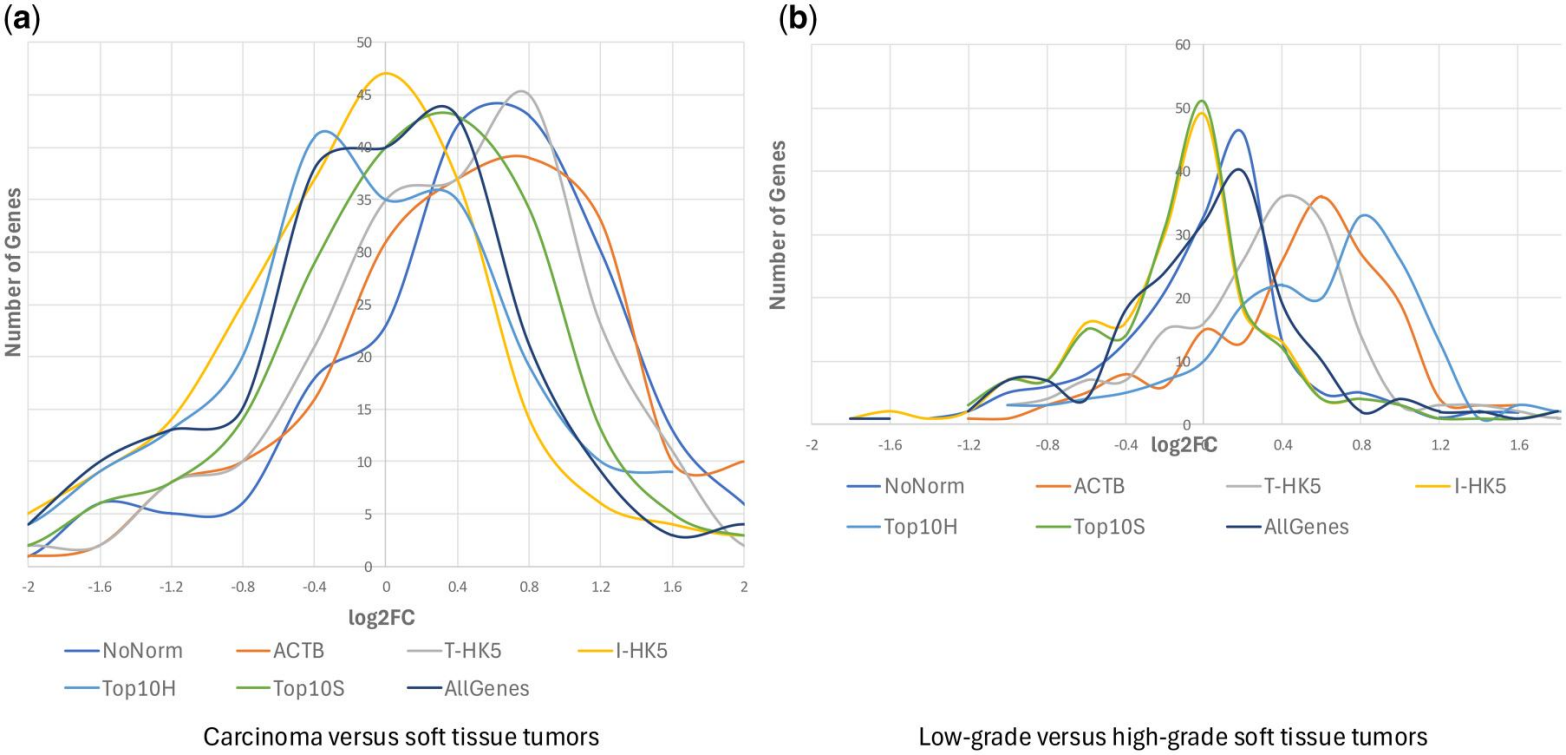
Effect on centralization of different normalization methods for 21 tumor samples (panel 2). The distribution of log2FC values generated by DESeq2 after read counting by featureCounts and normalization of read counts by six different methods. (a) Test set included 10 carcinomas and 11 soft-tissue tumors. Normalization methods shown are NoNorm, Top10H, ACTB, Top10S, T-HK5, AllGenes, and I-HK5, with abbreviations defined in text profiled by panel 2. (b) Effects for a distinct set of 30 low-grade and 37 high-grade soft-tissue tumors, graded according to French Federation of Cancer Centers Sarcoma Group (FNCLCC) grading system (Amin *et al*. 2017).

Using DESeq2 to compare gene expression in distinct tumor groups, the distributions of all the logFC values were calculated for downregulated and upregulated genes in carcinoma as compared to soft-tissue tumors in assay 2 (Fig. 2a). The distribution of the logFC values of upregulated genes was highly biased without normalization likely influenced by a small set of highly expressed genes related to extracellular matrix production and other general cellular processes. Normalization using *ACTB* or multiple housekeeping genes (T-HK5) exaggerated this effect. Normalization using Top10H better highlighted differences among down-regulated genes. Normalization using AllGenes and Top10S better centralized the distribution of the logFC values for upregulated genes, whereas the I-HK5 method centralized the distribution of the logFC values for both upregulated and downregulated genes.

To assess the effects of normalization strategies on determining tumor grade by gene expression patterns, the distribution of the logFC values of the read counts was compared to a set of low-grade soft-tissue tumors and high-grade sarcomas in assay 1 (Fig. 2b). Without normalization, upregulated genes biased the distribution as did AllGenes whereas *ACTB* or the T-HK5 and Top10H methods improved the separation between the groups. I-HK5 and Top10S produced better centralization of the distribution. Possibly due to effects on minimizing differences simply due to tumor cellularity, normalization using I-HK5 and Top10S produced excellent results for this assay indication.

### 3.3 Effect of expression normalization strategies on tumor type distinction

**Figure 3:**
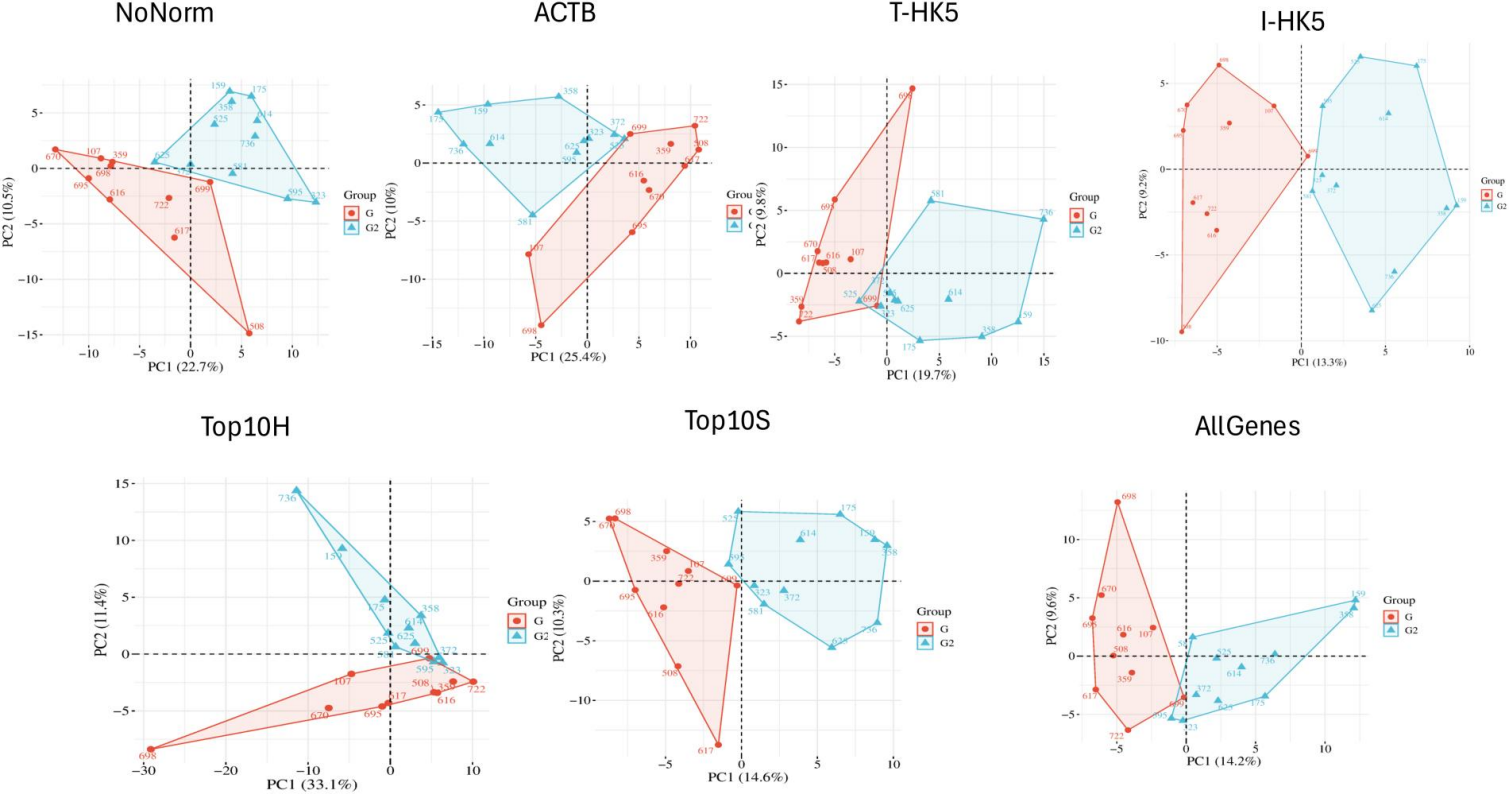
Effect on PCA clustering of different normalization methods comparing soft-tissue tumors and carcinomas. DGE and PCA were performed as in Section 2 for 10 carcinomas (red dots) and 11 soft-tissue tumors (light blue triangles) profiled by panel 2, with read counting by featureCounts and normalization of reads by the indicated method. The same 21 samples were used as in Fig. 2a. Method abbreviations are defined in the text.

An important goal of normalization strategies in targeted tumor RNA-seq assays is to enhance accuracy and separation of tumor type clusters to improve mapping of samples of unknown lineage. Several unsupervised clustering methods were compared, including PCA, t-SNE, and heatmap-clustering. By PCA, the I-HK5 and Top10S methods enhanced the separation of PCA clusters for the same test set of carcinomas and sarcomas, with I-HK5 showing the best separation (Fig. 3). Although a heatmap of the same data did highlight the tumor groups, the complicated display did not easily facilitate expression pattern of new samples during a quick review (not shown). With the I-HK5 normalization method, there was no obvious clustering with t-SNE for the carcinoma versus soft tissue diagnostic indication (not shown). Similar results are seen for the separate test set of low-grade and high-grade tumors (not shown).

**Figure 4:**
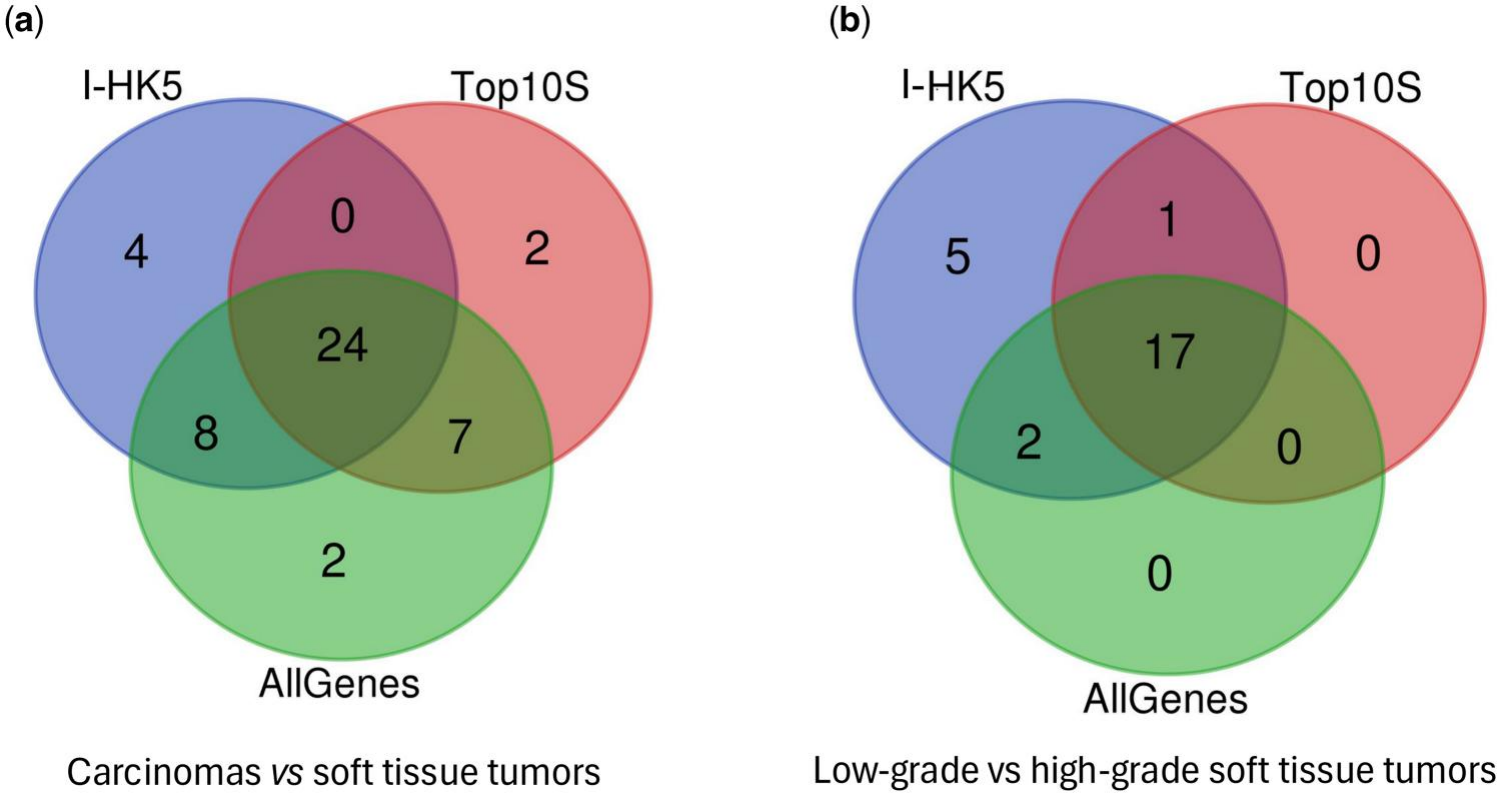
Comparison of normalization methods on outlier gene identification. DGE was performed as in Section 2, after read counting by featureCounts and normalization of reads by the indicated method, including I-HK5, Top10S, or using all genes (AllGenes) for normalization. Venn diagrams illustrate the number of shared and distinct significantly expressed genes in each category for these three normalization methods for (a) carcinoma versus soft-tissue tumors as in Figs 2a and 3 and (b) a separate set of low-grade versus high-grade soft-tissue tumors, as in Table S1, available as supplementary data at *Bioinformatics Advances* online.

The ability to identify similar or distinct genes sets was also assessed in DGE using the different normalization methods. For the same cases as in Figs 2a and 3, most of the differential carcinoma versus sarcoma genes were shared between the I-HK5, Top10S, and AllGenes methods (Fig. 4). However, I-HK5 displayed more additional significant genes as compared to the other 2 approaches.

**Figure 5:**
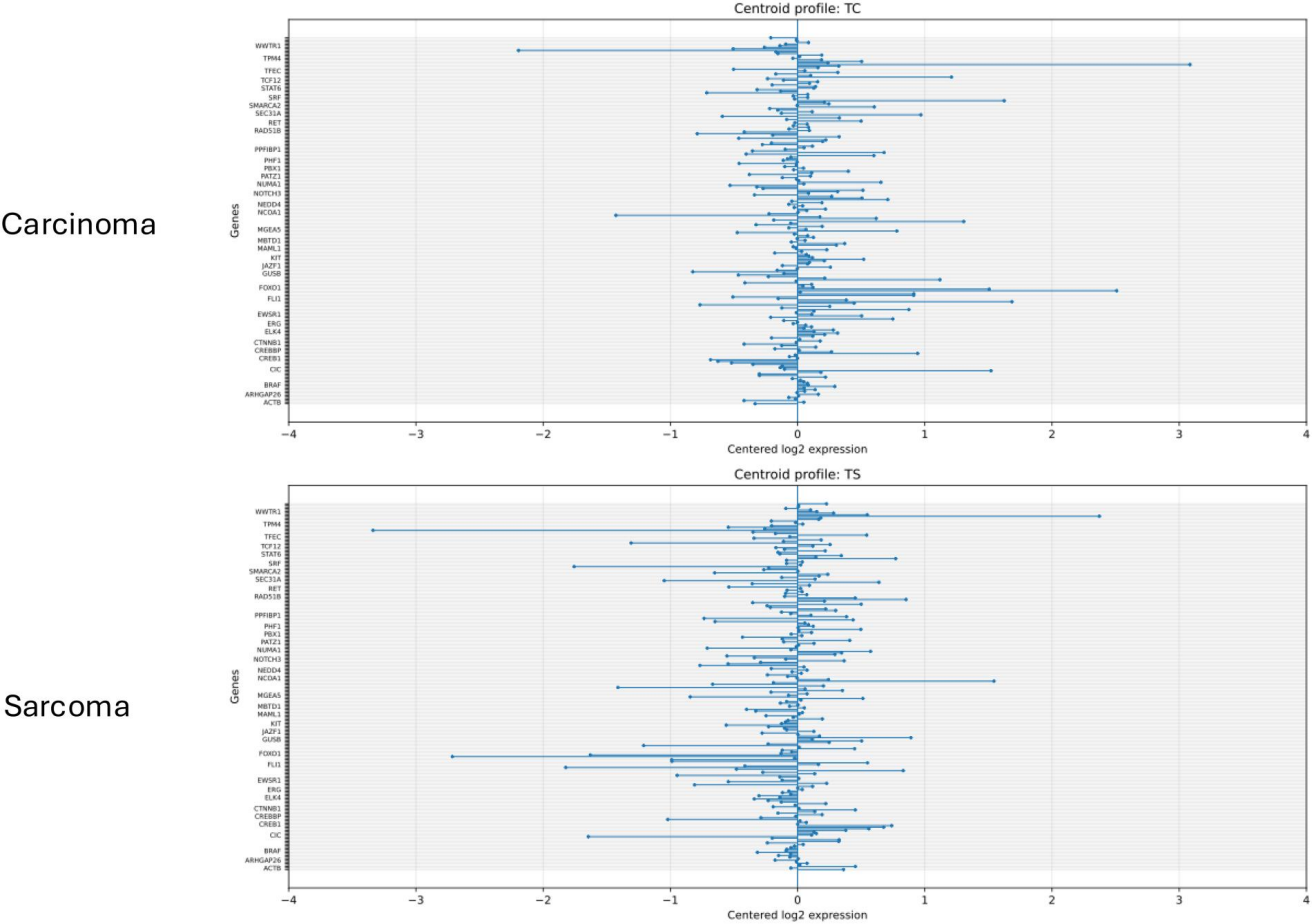
Mean centroid analysis of carcinomas (top) and sarcomas (bottom). DGE using panel 2, featureCounts and the HK5 normalization methods were performed on a 90-tumor training set of straightforward carcinomas and sarcomas. The mean centroid pattern for each of the 222 expressed genes for carcinoma (top) and sarcoma (bottom) are displayed with the names of the most significant genes shown for each group. The same 21 samples as in Figs 2a and 3 were called as carcinoma or sarcoma based on these centroids (see text for details).

After evaluating several different supervised approaches including centroids and logistic regression, we generated outputs of DGE from panel 2 following featureCounts enumeration and HK5 normalization using the mean centroid method. A training set of 90 tumors comprising a range of typical carcinoma and sarcomas were used to generate mean group centroids for each of the 222 expressed genes (Fig. 5). These centroids showed accuracy of 81% (8/10 carcinoma and 9/11 sarcoma) when assessing the same 21 cases presented in Figs 2 and 3.

### 3.4 Comparison of targeted RNA-seq outputs with total RNA-seq in challenging tumor set

**Figure 6:**
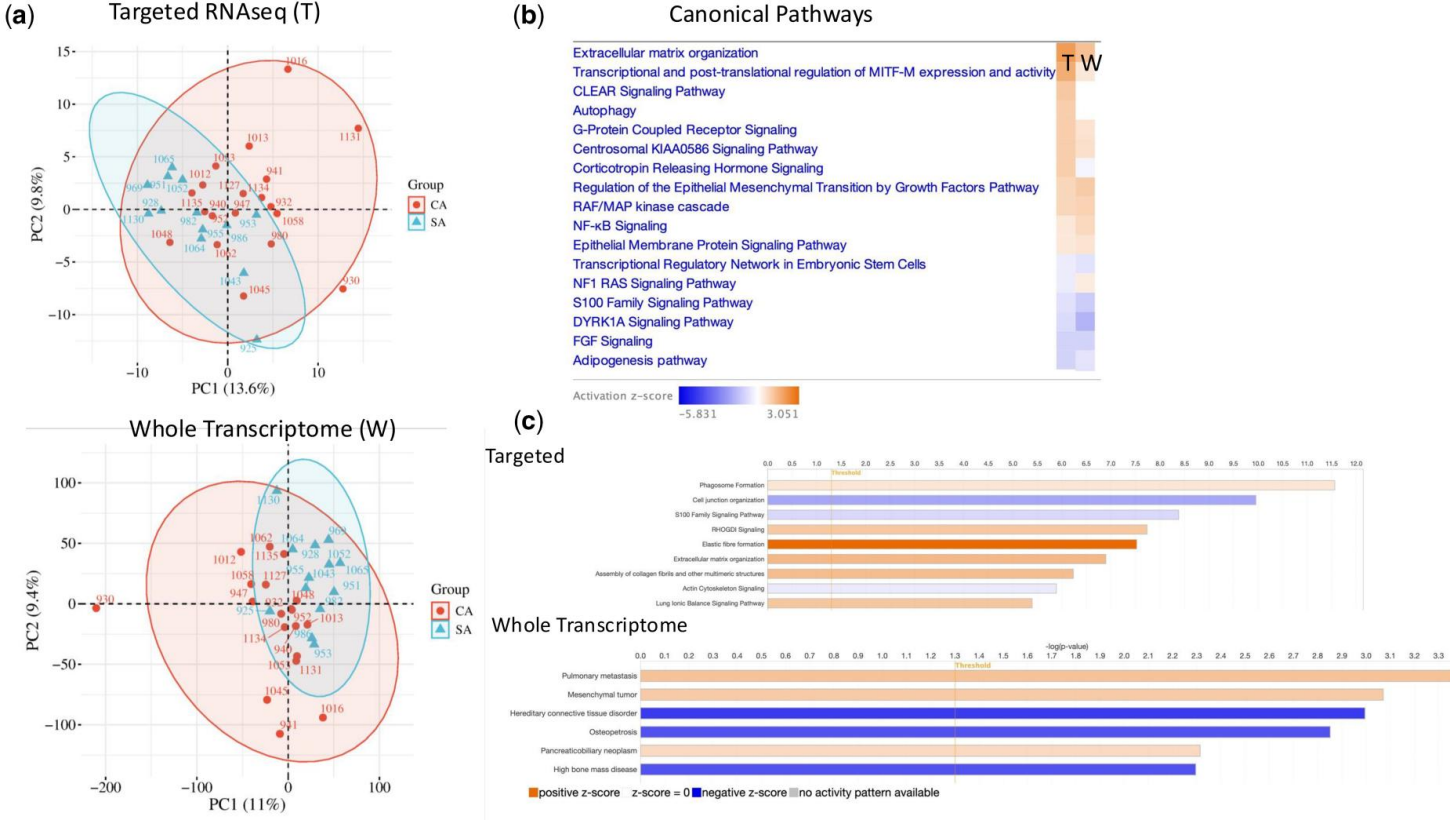
Validation of the optimized targeted RNA-seq pipeline by comparison with total RNAseq data. DESeq2 was performed using the targeted panel 2 (T) or whole transcriptome (W) on 32 diagnostically challenging malignant tumors (19 classified as carcinomas, 13 sarcomas) and compared with whole transcriptome performed on the same RNA. (a) PCA pattern shows a similar separation of carcinomas (CA, red dots) and sarcomas (SA, blue triangles) with targeted panel and whole transcriptome. (b) Ingenuity pathway analysis (IPA) shows similar canonical pathways that are upregulated (red) or downregulated (blue) in the carcinomas as compared to sarcomas. (c) Correlated disease states identified by IPA among the carcinomas (red) and sarcomas (blue); however, are largely distinct between targeted panel and whole RNA-seq.

To validate the DGE output of the targeted panel 2, we compared the outputs with those obtained by total RNA-seq on a diagnostically relevant set of 32 poorly differentiated tumors (19 likely carcinoma, 13 likely sarcoma). Although the number of expressed genes across all samples was vast differently (226 versus 20 218; Table 4), expression levels of the 32 shared expressed genes was high (*r* = 0.95) indicating similar quality studies. The most significantly differentially expressed genes between carcinoma and sarcoma cases (at adjusted *P* value <.05 level) were very similar for the shared genes (Table S5, available as supplementary data at *Bioinformatics Advances* online). The mapping of these cases into carcinoma and sarcoma clusters was also similar by PCA (Fig. 6a). The top differentially upregulated or downregulated canonical pathways by IPA analysis were also similar (Fig. 6b). However, most of the disease states differentially regulated in sarcomatoid carcinoma and sarcoma, as identified by IPA, were distinct when the transcriptome data was compared to the targeted panel (Fig. 5c and Table S6, available as supplementary data at *Bioinformatics Advances* online). Although S100 family signaling was a shared upregulated pathway in sarcomas by both analyses, the targeted panel identified more specific signaling pathways in the carcinoma group.

**Table 4:** Comparison of expressed genes in optimized outputs for targeted RNAseq (panel 2) and full RNAseq for a test set of carcinoma and soft tissue tumors.

Datasets	Total genes	Differentially expressed genes^a^	Differentially expressed genes (%)
**Total RNA-seq**	20 218	2591	12.8
**Targeted RNA-seq**	226	47	14.2

a Criteria for the significantly differentially expressed genes selected were adjusted *P* value of .05 and log2FC cutoffs of ⩽−0.7 and ⩾0.7.

## 4 Discussion

Many of the large number of targeted RNA-seq gene fusion assays performed routinely in tumors have not been utilized for DGE to date. Given that only a small minority of tumors will have oncogenic gene fusions (Heyer *et al.* 2019), this results in most fusion assays contributing little to the diagnostic cancer workup. Extending the approach used in tumor immunostaining, we hypothesized that targeted tumor panels that include only a small number of lineage-associated genes along with the set of genes implicated in solid tumor-associated gene fusions and mutations can have useful diagnostic discriminatory power. Using clinical-grade ∼200-gene RNA-seq assays originally developed for gene fusion detection, we have systematically evaluated their suitability for tumor lineage assessment and grading. The best methods for read counting for pipeline optimization and data normalization using DGE were studied, with outputs for supervised (mean centroid) and unsupervised (PCA) clustering displayed for carcinomas and sarcomas.

The output of the optimized targeted panel pipeline was then compared to whole transcriptome RNA-seq for more challenging tumors, likely representing various types of carcinoma or sarcoma. Using the 230-gene fusion assay containing several lineage-specific markers, the targeted panel showed similar clustering of tumor samples and some shared features by pathway analysis as the whole transcriptome results. Thus, fusion panels with just a few added genes may encode significant diagnostic power in fusion-negative cases. This is likely partly due to some fusion genes also driving oncogenesis in similar tumors by mutational upregulation or transcriptionally-mediated overexpression (Mertens *et al.* 2015, Franco *et al.* 2022, Bolotin *et al.* 2023). An example is the presence of estrogen receptor (ER) fusions as well as mutations and structural rearrangements producing ER overexpression in breast cancer (Hancock *et al.* 2024). The addition to the panel of cell lineage-specific transcription factors that are both mutational targets and drive cellular differentiation likely also increases diagnostic power. Examples include *MYOD1* expression driving skeletal muscle differentiation in both wild-type and MYOD1-mutated rhabdomyosarcoma (Avenarius *et al.* 2024) and increased levels of melanocyte differentiation genes in clear cell tumors related to melanoma (Segal *et al.* 2003).

Given the clinical impact of molecular tumor classification, RNA-seq analysis methods need to be carefully validated, and outputs need to be relatively easy to interpret. Since targeted RNA-seq often includes some targets whose expression is highly variable and sensitive to sample quality, certain wet lab and pipeline methods may produce incomplete or inaccurate results. The selection of a hybrid capture methodology here reflects the common use of this method to achieve better read balance across gene targets. Amplicon-based sequencing methods are also employed for RNA sequencing but the greater variability often observed in gene coverage in such panels that include many genes may require additional quality control. For these assays, a focus on exon imbalance metrics (Goytain *et al.* 2023, Gaziev *et al.* 2025) and different normalization strategies may be useful. Another analytic option in primary sequence analysis is the use of pseudoaligners against the transcriptome (as opposed to the genome) such as Salmon or Kallisto (Yi *et al.* 2018). These tools provide rapid alignment outputs but also introduce additional complexity in preprocessing and may result in lower read depths in tumor analysis (Acedo-Terrades *et al.* 2025). However, these tools can also identify differential transcript patterns that may have diagnostic utility.

Many read counting algorithms have been developed and used for whole transcriptome RNA-seq. As shown in Table S4, available as supplementary data at *Bioinformatics Advances* online, different read counting methods vary in speed and performance. Our prior method, the Samtools built-in program “view” calculates sequence reads in the targeted regions from alignment BAMs accurately when validated with the proper option settings (Danecek *et al.* 2021). But the long processing times can cause delays in clinical reporting. CuffDiff is another widely used software tool for DGE analysis in whole transcriptome RNA-seq (Trapnell *et al.* 2012). It has a function to generate the number of sequence reads per gene in a sample. HTSeq-count is another widely used tool (Anders *et al.* 2015). BEDTools/coverageBed with the “-count” option is another option that allows multiple samples to be analyzed in parallel (Quinlan and Hall 2010). A previous study compared six methods of read counting for RNA-seq data and reported HTSeq-count was superior (Corchete *et al.* 2020). However, that study did not include coverageBed, Samtools-view, or featureCounts, which is optimized for efficient chromosome hashing and feature blocking techniques and parallel analysis (Liao *et al.* 2014). They also did not perform assessments of speed performance, which is critical for clinical applications.

Comparing speed, precision, accuracy, and ease of use, we found that featureCounts and coverageBed had a significant advantage over other methods in output time, with featureCounts being the fastest. By statistical measures of accuracy and precision, featureCounts, coverageBed, HTSeq-count, and Samtools-view had highly similar outputs. However, we found that CuffDiff underestimates sequence reads significantly which was traced to the limitations in the maximum number of reads in the buffer of the software allowance which produces trimming of some reads from larger genes. Given that featureCounts and HTSeq-count require conversion of a BED into a GTF file, the ability of coverageBed to directly consume a BAM file is a great feature for smaller panels where the BAM files are not very large.

When small gene panels are employed for DGE, minimizing skewing of the overall gene expression distribution (or centralization) is critical. To find the optimal normalization method of sequence reads for DGE from small RNA-seq fusion panels, we compared multiple common employed strategies and a lab/assay-specific method. Use of a single housekeeping gene such (*ACTB*) or multiple housekeeping genes performed poorly in reducing intra-tumor group variation. In contrast, the empirically determined five most consistently and stably expressed genes (I-HK5) across the panels and tumors typically seen in our laboratory performed well. This method reduced intragroup variances significantly similar to using all genes or the top 10 highly expressed genes. The significantly expressed genes identified by DGE were also mostly similar with these three methods. The I-HK5 approach also improved centralization of log2FC values after DGE and improved the separation of PCA clusters for the carcinoma versus sarcoma and tumor grading indications. These findings emphasize that assessment of different centralization/normalization methods, especially for more targeted RNA-seq panel, should be included in the validation of each new assay with sample sets tuned for the specific clinical applications.

Consideration of different data display methods is also critical given the limited time available for analysis for clinical assays. For tumor lineage analysis, we employed the supervised clustering nearest centroid method to assess the categories of carcinoma and sarcoma. With a 90-sample training set, we achieved an accuracy of ∼80% in a small test set. This was compared with clustering observed with unsupervised clustering methods. When mapping expression patterns of new/unknown tumors to an existing dataset, we found that PCA is the best sample unsupervised clustering method. By displaying typical tumor groups clearly (e.g. carcinoma versus sarcoma, high-grade versus low-grade), it can efficiently identify when an unknown sample maps well within a group as opposed to being an outlier where the study may be non-informative. Although heatmaps are a useful visualization method when tumor expression patterns are highly similar (e.g. cultured tumor cells exposed to different drug doses), it is difficult to visualize tumor-group differences when a new diagnostic sample is introduced.

Using a more challenging carcinoma versus sarcoma tumor set, we noted that an optimized targeted panel with some lineage-specific genes included (panel 2) showed equivalent separation by PCA compared to whole RNA-seq. In addition, many of the top tumor class gene discriminators were shared by the two assays. This similar result was further noted by IPA comparison of the top differentially regulated canonical signaling pathways. However, disease states mapping by IPA among the carcinomas and sarcomas was largely distinct between targeted panel and total RNA-seq. This was likely due to the effects of genes not present in the targeted panel that provided additional subclass differentiation.

In summary, we identified featureCounts as the optimal read counting method and the five assay-specific most stable genes (I-HK5) as the best method for normalization prior to DESeq2. Optimization of the bioinformatic pipeline allows effective use of clinical-grade targeted RNA-seq panels originally designed for fusion analysis. Comparable utility may be obtained for targeted panels as compared to full RNA-seq when assessing a group of tumors for cell lineage. For any given tumor, comparison of the expression pattern for these critical genes against those seen in major classes of tumors (e.g. carcinoma, melanoma, and soft-tissue tumors) can help determine site of origin when other biomarkers and microscopic studies are not informative. These comparisons provide a model for validating these methods for routine clinical use through systematic assessment of analytic tools and incremental assay design.

## Supplementary Material

vbag197_Supplementary_Data

## Data Availability

The data underlying this article are available from the corresponding author on reasonable request.

## References

[vbag197-B1] Acedo-Terrades A , Perera-Bel J, Nonell L. The importance of data transformation in RNA-Seq preprocessing for bladder cancer subtyping. BMC Res Notes 2025;18:61. 39930545 10.1186/s13104-025-07138-x PMC11812149

[vbag197-B2] Aisner DL , Gocke CD, Jones D et al The genomics organization for academic laboratories (GOAL): a vision for a genomics future for academic pathology. Acad Pathol 2023;10:100090. 37583476 10.1016/j.acpath.2023.100090 PMC10424130

[vbag197-B3] Amin MB , Edge SB, Greene FL et al AJCC Cancer Staging Manual. 8th edn. New York: Springer, 2017.

[vbag197-B4] Anders S , Pyl PT, Huber W. HTSeq—a python framework to work with high-throughput sequencing data. Bioinformatics 2015;31:166–9. 25260700 10.1093/bioinformatics/btu638 PMC4287950

[vbag197-B5] Avenarius MR , Patton A, Mohamed N et al Integrated molecular profiling of rhabdomyosarcoma subtypes by targeted RNA-seq. medRxiv, 10.1101/2024.10.11.24315314, 2024, preprint: not peer reviewed.

[vbag197-B6] Bolotin E , Melamed D, Livnat A. Genes that are used together are more likely to be fused together in evolution by mutational mechanisms: a bioinformatic test of the used-fused hypothesis. Evol Biol 2023;50:30–55. 36816837 10.1007/s11692-022-09579-9 PMC9925542

[vbag197-B7] Bushel PR , Ferguson SS, Ramaiahgari SC et al Comparison of normalization methods for analysis of TempO-Seq targeted RNA sequencing data. Front Genet 2020;11:594. 32655620 10.3389/fgene.2020.00594 PMC7325690

[vbag197-B8] Capone I , Bozzi F, Dagrada GP et al Targeted RNA-sequencing analysis for fusion transcripts detection in tumour diagnostics: assessment of bioinformatic tools reliability in FFPE samples. Explor Target Antitumor Ther 2022;3:582–97. 36338518 10.37349/etat.2022.00102 PMC9630092

[vbag197-B9] Corchete LA , Rojas EA, Alonso-López D et al Systematic comparison and assessment of RNA-seq procedures for gene expression quantitative analysis. Sci Rep 2020;10:19737. 33184454 10.1038/s41598-020-76881-x PMC7665074

[vbag197-B10] Curion F , Handel AE, Attar M et al Targeted RNA sequencing enhances gene expression profiling of ultra-low input samples. RNA Biol 2020;17:1741–53. 32597303 10.1080/15476286.2020.1777768 PMC7746246

[vbag197-B11] Dabney AR , Storey JD. Optimality driven nearest centroid classification from genomic data. PLoS One 2007;2:e1002. 17912341 10.1371/journal.pone.0001002 PMC1991588

[vbag197-B12] Danecek P , Bonfield JK, Liddle J, et al Twelve years of SAMtools and BCFtools. GigaScience 2021;10:giab008. 33590861 10.1093/gigascience/giab008 PMC7931819

[vbag197-B13] de Brito MW , de Carvalho SS, Mota MB et al RNA-seq validation: software for selection of reference and variable candidate genes for RT-qPCR. BMC Genomics 2024;25:697. 39014352 10.1186/s12864-024-10511-y PMC11251314

[vbag197-B14] Dobin A , Davis CA, Schlesinger F et al STAR: ultrafast universal RNA-seq aligner. Bioinformatics 2013;29:15–21. 23104886 10.1093/bioinformatics/bts635 PMC3530905

[vbag197-B15] Evans C , Hardin J, Stoebel DM. Selecting between-sample RNA-seq normalization methods from the perspective of their assumptions. Brief Bioinform 2018;19:776–92. 28334202 10.1093/bib/bbx008 PMC6171491

[vbag197-B16] Franco AT , Ricarte-Filho JC, Isaza A et al Fusion oncogenes are associated with increased metastatic capacity and persistent disease in pediatric thyroid cancers. J Clin Oncol 2022;40:1081–90. 35015563 10.1200/JCO.21.01861 PMC8966969

[vbag197-B17] Fu C , Marczyk M, Samuels M et al Targeted RNA-seq assay incorporating unique molecular identifiers for improved quantification of gene expression signatures and transcribed mutation fraction in fixed tumour samples. BMC Cancer 2021;21:114. 33541297 10.1186/s12885-021-07814-8 PMC7860187

[vbag197-B18] Gaziev I , Khristichenko A, Luppov D et al Accurate RET fusion detection in solid tumors using RNA sequencing coverage imbalance analysis. Int J Mol Sci 2025;26:11300. 41373459 10.3390/ijms262311300 PMC12692729

[vbag197-B19] Goytain A , Chang KT, Goh JY et al Diagnosis of fusion-associated sarcomas by exon expression imbalance and gene expression. J Mol Diagn 2023;25:121–31. 36503147 10.1016/j.jmoldx.2022.11.004

[vbag197-B20] Hancock GR , Gertz J, Jeselsohn R et al Estrogen receptor alpha mutations, truncations, heterodimers, and therapies. Endocrinology 2024;165:bqae051. 38643482 10.1210/endocr/bqae051 PMC11075793

[vbag197-B21] He B , Sun H, Bao M et al A cross-cohort computational framework to trace tumor tissue-of-origin based on RNA sequencing. Sci Rep 2023;13:15356. 37717102 10.1038/s41598-023-42465-8 PMC10505149

[vbag197-B22] Heyer EE , Deveson IW, Wooi D et al Diagnosis of fusion genes using targeted RNA sequencing. Nat Commun 2019;10:1388. 30918253 10.1038/s41467-019-09374-9 PMC6437215

[vbag197-B23] Johnson L. github: pure Java implementation of Van Der Maaten and Hinton’s t-SNE clustering algorithm. 2023. Version 2.6.4. https://github.com/lejon/T-SNE-Java (15 February 2026, date last accessed).

[vbag197-B24] Li D , Zand MS, Dye TD, et al An evaluation of RNA-seq differential analysis methods. PLoS One 2022;17:e0264246. 36112652 10.1371/journal.pone.0264246 PMC9480998

[vbag197-B25] Li J , Fu C, Speed TP et al Accurate RNA sequencing from formalin-fixed cancer tissue to represent high-quality transcriptome from frozen tissue. JCO Precis Oncol 2018;2:1–9. 10.1200/PO.17.00091 PMC5976456 29862382

[vbag197-B26] Liao Y , Gordon K, Smyth GK et al featureCounts: an efficient general-purpose program for assigning sequence reads to genomic features. Bioinformatics 2014;30:923–30. 24227677 10.1093/bioinformatics/btt656

[vbag197-B27] Love MI , Huber W, Anders S. Moderated estimation of fold change and dispersion for RNA-seq data with DESeq2. Genome Biol 2014;15:550. 25516281 10.1186/s13059-014-0550-8 PMC4302049

[vbag197-B28] Maza E , Frasse P, Senin P et al Comparison of normalization methods for differential gene expression analysis in RNA-Seq experiments: a matter of relative size of studied transcriptomes. Commun Integr Biol 2013;6:e25849. 26442135 10.4161/cib.25849 PMC3918003

[vbag197-B29] Mertens F , Johansson B, Fioretos T et al The emerging complexity of gene fusions in cancer. Nat Rev Cancer 2015;15:371–81. 25998716 10.1038/nrc3947

[vbag197-B30] Nakamura H , Kukita Y, Wakamatsu T et al Targeted RNA sequencing enhances the integrated diagnosis of bone and soft tissue tumors. Hum Pathol 2026;168:106036. 41506608 10.1016/j.humpath.2026.106036

[vbag197-B31] QIAGEN Digital Insights. Ingenuity Pathway Analysis. 2026. https://digitalinsights.qiagen.com/products-overview/discovery-insights-portfolio/analysis-and-visualization/qiagen-ipa (15 February 2026, date last accessed).

[vbag197-B32] Quinlan AR , Hall IM. BEDTools: a flexible suite of utilities for comparing genomic features. Bioinformatics 2010;26:841–2. 20110278 10.1093/bioinformatics/btq033 PMC2832824

[vbag197-B33] Rau A , Gallopin M, Celeux G et al Data-based filtering for replicated high-throughput transcriptome sequencing experiments. Bioinformatics 2013;29:2146–52. 23821648 10.1093/bioinformatics/btt350 PMC3740625

[vbag197-B34] Robinson JT , Thorvaldsdóttir H, Winckler W et al Integrative genomics viewer. Nat Biotechnol 2011;29:24–6. 21221095 10.1038/nbt.1754 PMC3346182

[vbag197-B35] Segal NH , Pavlick AC, Maimon A et al Classification of clear-cell sarcoma as a subtype of melanoma by genomic profiling. J Clin Oncol 2003;21:1775–81. 12721254 10.1200/JCO.2003.10.108

[vbag197-B36] Tang D , Chen M, Huang X, et al SRplot: a free online platform for data visualization and graphing. PLoS One 2023;18:e0294236. 37943830 10.1371/journal.pone.0294236 PMC10635526

[vbag197-B37] Tibshirani R , Hastie T, Narasimhan B et al Diagnosis of multiple cancer types by shrunken centroids of gene expression. Proc Natl Acad Sci 2002;99:6567–72. 12011421 10.1073/pnas.082099299 PMC124443

[vbag197-B38] Trapnell C , Roberts A, Goff L et al Differential gene and transcript expression analysis of RNA-seq experiments with TopHat and cufflinks. Nat Protoc 2012;7:562–78. 22383036 10.1038/nprot.2012.016 PMC3334321

[vbag197-B39] Uhrig S , Ellermann J, Walther T et al Accurate and efficient detection of gene fusions from RNA sequencing data. Genome Res 2021;31:448–60. 33441414 10.1101/gr.257246.119 PMC7919457

[vbag197-B40] Wang M , Klevebring D, Lindberg J et al Determining breast cancer histological grade from RNA-sequencing data. Breast Cancer Res 2016;18:48. 27165105 10.1186/s13058-016-0710-8 PMC4862203

[vbag197-B41] Wei IH , Shi Y, Jiang H et al RNA-Seq accurately identifies cancer biomarker signatures to distinguish tissue of origin. Neoplasia 2014;16:918–27. 25425966 10.1016/j.neo.2014.09.007 PMC4240918

[vbag197-B42] Yi L , Liu L, Melsted P, et al A direct comparison of genome alignment and transcriptome pseudoalignment. *BioRxiv*, 10.1101/444620, 2018, preprint: not peer reviewed.

[vbag197-B43] Zhao Y , Li MC, Konaté MM et al TPM, FPKM or normalized counts? A comparative study of quantification measures for the analysis of RNA-seq data from the NCI patient-derived models repository. J Transl Med 2021;19:269. 34158060 10.1186/s12967-021-02936-w PMC8220791

