# Supplementary material for: Optimizing bioinformatic workflows to extract clinically usable gene expression data from targeted tumor RNA sequencing panels: comparison with total RNA-seq in cancer samples
Source: Bioinform Adv. 2026 Jul 13;6(1):vbag197. doi: 10.1093/bioadv/vbag197 (PMC13418193; doi:10.1093/bioadv/vbag197)
Supplement: vbag197_Supplementary_Data [file vbag197_supplementary_data.docx]

**Table S1.** Samples used for clustering and normalization study.

| *Group* | *Samples IDs as displayed on Figure 3* | *Panel* | |
| --- | --- | --- | --- |
| Low-grade Sarcoma | 225, 226, 227, 228, 244, 258, 259, 281, 289, 290, 300, 304, 305, 308, 311, 313, 314, 317, 322, 325, 330, 345, 352, 353, 363, 367, 370, 375, 381, 392 | | 1 |
| High-grade Sarcoma | 395, 400, 409, 414, 415, 416, 421, 230, 242, 252, 253, 265, 277, 283, 295, 307, 309, 312, 323, 348, 355, 357, 358, 362, 364, 369, 372, 382, 385, 389, 393, 399, 401, 403, 417, 427, 429 | | 1 |
| Carcinoma | 107, 359, 695, 698, 616, 699, 670, 617, 508, 722 | | 2 |
| Soft tissue tumors | 595, 581, 372, 625, 525, 159, 358, 175, 736, 614, 323 | | 2 |

**Table S2.** Depth of coverage in samples sequenced by both targeted and total RNA-seq.

| *Sample ID* | *Tumor type* | *Targeted RNA-seq* | | *Whole RNA-seq* | |
| --- | --- | --- | --- | --- | --- |
|  | Reads per segment > | Raw reads | Normalized reads | Raw reads | Normalized reads |
| 1012 | PD carcinoma | 87839 | 198673 | 1745 | 1578 |
| 1013 | PD carcinoma | 129635 | 213509 | 644 | 1191 |
| 1016 | Basaloid tumor/carcinoma | 161118 | 239873 | 801 | 1301 |
| 1045 | High-grade tumor/neuroendocrine | 197225 | 149129 | 1031 | 1075 |
| 1048 | Spindle cell tumor/carcinoma | 987505 | 867767 | 1737 | 1684 |
| 1053 | Acinic tumor/carcinoma | 82657 | 151362 | 873 | 1223 |
| 1058 | Clear cell tumor | 287568 | 237835 | 1116 | 1560 |
| 1062 | Mixed carcinoma/renal origin | 378336 | 352092 | 753 | 1738 |
| 1127 | PD carcinoma | 239805 | 282256 | 1795 | 1499 |
| 1131 | PD Carcinoma | 203396 | 298603 | 1002 | 1295 |
| 1134 | PD Carcinoma | 272458 | 294257 | 903 | 1329 |
| 1135 | Sarcomatoid carcinoma | 192147 | 397029 | 725 | 1430 |
| 930 | PD carcinoma | 132128 | 279143 | 680 | 1980 |
| 932 | PD carcinoma | 143618 | 301958 | 1270 | 1396 |
| 940 | PD carcinoma | 300035 | 233758 | 1559 | 1278 |
| 941 | Neuroendocrine/small cell carcinoma | 152622 | 183659 | 793 | 1142 |
| 947 | PD carcinoma | 148977 | 181806 | 965 | 1343 |
| 952 | PD carcinoma | 469254 | 264922 | 367 | 1258 |
| 980 | PD carcinoma | 219665 | 261330 | 667 | 1196 |
| 1043 | Epithelioid sarcoma | 519113 | 327043 | 1627 | 1573 |
| 1052 | Epithelioid leiomyosarcoma | 302297 | 490324 | 1077 | 1516 |
| 1064 | Epithelioid neoplasm/sarcoma | 783599 | 749294 | 1167 | 1706 |
| 1065 | Spindle cell neoplasm/sarcoma | 762451 | 770452 | 1355 | 1591 |
| 1130 | Dedifferentiated dermal sarcoma | 315071 | 326981 | 1030 | 1721 |
| 925 | Dedifferentiated liposarcoma | 687536 | 454461 | 1802 | 1366 |
| 928 | Undifferentiated pleomorphic sarcoma | 533397 | 279169 | 2108 | 1457 |
| 951 | Myxoid neoplasm/sarcoma | 438755 | 288123 | 1441 | 1333 |
| 953 | Epithelioid neoplasm/leiomyosarcoma | 447982 | 336311 | 866 | 1234 |
| 955 | Spindle cell neoplasm/sarcoma | 1179928 | 934164 | 823 | 1456 |
| 969 | Epithelioid neoplasm/myxofibrosarcoma | 391959 | 311698 | 2355 | 1477 |
| 982 | Malignant peripheral nerve sheath tumor | 527682 | 315909 | 1007 | 1197 |
| 986 | Malignant peripheral nerve sheath tumor | 754581 | 786338 | 1159 | 1373 |
| Mean coverages | | 388448.1 | 367475.9 | 1163.8 | 1421.8 |

**Table S3.** List of top 10 genes with smallest CVs tested by either assay 1 or assay 2 (top 5 genes shaded).

| Set | Top 10 most stably expressed genes for each sample set | Panel-group |
| --- | --- | --- |
| 1 | *CREBBP, BRAF, BRD4, ATF1, CDK4, CIC, CREB1, CLTC, BCORL1, ASPSCR1* | 2-run1 |
| 2 | *ASPSCR1, BRAF, CLTC, BRD4, ACTB, CREB1, ATF1, CAMTA1, CDK4, CREBBP* | 2-run3 |
| 3 | *CREBBP, ATF1, CLTC, BRAF, CREB1, BRD4, BCOR, ASPSCR1, CDK4, CREB3L2* | 2-run4 |
| 4 | *CREBBP, CREB1, BRAF, CLTC, BRD4, CAMTA1, BCORL1, ATF1, ASPSCR1, BRD3* | 2-run5 |
| 5 | *ATF1, CREBBP, CREB1, BRAF, BRD4, BRD3, BCORL1, BCOR, CDK4, CREB3L1* | 2-run6 |
| 6 | *CREBBP, CRTC3, ATRX, BCORL1, CAMTA1, BRAF, CREB1, ATF1, BRD4,BCOR* | 1-carcinomas & non-neoplastic epithelial |
| 7 | *BRAF, ATF1, CREBBP, ATRX, CARS, CREB1, ACTB, CREM, BRD4,CRTC1* | 1: all soft tissue |
| 8 | *CREBBP, BRAF, ATF1, BRD4, CREB1, ATRX, ACTB, BRD3, CIC, CAMTA1* | 1: soft tissue & carcinoma combined |
| 9 | *CREBBP, ATF1, ASPSCR1, BRD4, BRAF, CREB1, ACTB, CIC, BRD3, CDH11* | 1: Carcinomas versus other poorly differentiated |
| 10 | *CREBBP, BRAF, ATF1, BRD4, CREB1, ACTB, CIC, ASPSCR1, BRD3, CREB3L2* | 1: soft tissue & non-neoplastic stromal tissues |

**Table S4.** Representative run time of the counting methods (10-sample run).

| *Counting Method* | *Run time* |
| --- | --- |
| Samtools-view | 6 hours |
| FeatureCounts | 2 minutes |
| HTSeq-count | 3 hours |
| CoverageBED | 9 minutes |
| CuffDiff | 4 hours |

**Table S5.** Shared significantly differentially genes using DESeq2 for targeted (T) and total RNA-seq (W) analysis between challenging carcinoma versus sarcoma cases (positive T-log2fc value).

| *Gene* | *T-BaseMean* | *T-log2fc* | *T-Adj_Pvalue* | *W-BaseMean* | *W-log2fc* | *W-Adj_Pvalue* |
| --- | --- | --- | --- | --- | --- | --- |
| *FGFR3* | 59925.4569 | -4.1438 | <0.0001 | 1295.6684 | -4.0473 | <0.0001 |
| *MYB* | 75609.0037 | -4.6381 | <0.0001 | 451.3362 | -3.5866 | <0.0001 |
| *FOXA1* | 43033.7619 | -4.7888 | <0.0001 | 963.1537 | -4.9744 | <0.0001 |
| *TMPRSS2* | 40541.335 | -6.1718 | <0.0001 | 447.9468 | -6.1329 | <0.0001 |
| *NKX2-1* | 2353.3977 | -7.5803 | <0.0001 | 57.2015 | -8.0463 | <0.0001 |
| *QKI* | 231602.8786 | 0.7366 | 0.0036 | 5888.2526 | 1.0224 | <0.0001 |
| *HAS2* | 46341.5531 | 2.115 | 0.0105 | 764.1334 | 3.6196 | <0.0001 |
| *TFEC* | 21268.3709 | 1.0349 | 0.0208 | 434.1639 | 1.5543 | <0.0001 |
| *CSF1* | 113781.581 | 1.6357 | <0.0001 | 3887.0143 | 1.6884 | 0.0001 |
| *COL6A3* | 4214311.185 | 2.2231 | 0.0002 | 75070.4201 | 2.2837 | 0.0002 |
| *ETV1* | 61358.5391 | 1.9368 | 0.0022 | 1021.6821 | 2.0819 | 0.0005 |
| *COL1A2* | 5508768.619 | 2.2127 | 0.0008 | 123118.9108 | 1.9597 | 0.0021 |
| *NOTCH2* | 821986.8394 | 0.9176 | 0.0116 | 14997.8865 | 0.9495 | 0.0023 |
| *FOXA2* | 4725.5723 | -4.2013 | 0.0036 | 124.8472 | -4.225 | 0.0024 |
| *FN1* | 12108270.46 | 1.9822 | 0.0084 | 211217.8499 | 2.2671 | 0.0026 |
| *PAX3* | 6627.3849 | 2.9413 | 0.0142 | 125.6044 | 2.109 | 0.0052 |
| *CDH11* | 308937.7461 | 1.5176 | 0.0125 | 4484.9399 | 1.6216 | 0.0069 |
| *VCL* | 375395.8772 | 0.8105 | 0.0154 | 6738.7134 | 0.8237 | 0.0069 |
| *GLI1* | 34812.1756 | 2.1904 | 0.0062 | 408.1723 | 1.8407 | 0.0072 |
| *PRRX1* | 102723.6524 | 1.6306 | 0.0125 | 3884.3553 | 1.8018 | 0.0074 |
| *VGLL3* | 41352.4971 | 1.584 | 0.0187 | 1738.1621 | 1.7829 | 0.008 |
| *WWTR1* | 134948.5214 | 1.0379 | 0.0154 | 3695.4338 | 1.1614 | 0.009 |
| *HMGA2* | 120275.5928 | 2.344 | 0.0187 | 1237.4033 | 2.5537 | 0.0106 |
| *FGFR1* | 325663.5221 | 1.5517 | 0.0142 | 7059.4409 | 1.6084 | 0.0114 |
| *SOX2* | 9641.3584 | -3.244 | 0.0063 | 256.1413 | -2.7398 | 0.0145 |
| *PDGFRA* | 250425.6639 | 1.5475 | 0.0226 | 4812.2195 | 1.6644 | 0.0145 |
| *ETV5* | 119253.0977 | 0.8871 | 0.0439 | 1441.4169 | 1.0753 | 0.0151 |
| *SDC4* | 79578.2804 | -1.2242 | 0.0301 | 4468.4981 | -1.242 | 0.0163 |
| *FGFR2* | 69635.6224 | -2.0686 | 0.0501 | 1613.9932 | -2.6397 | 0.0177 |
| *SQSTM1* | 459929.9321 | 0.8327 | 0.0291 | 11038.9687 | 0.7655 | 0.02 |
| *EZR* | 429798.8695 | -1.1102 | 0.0232 | 7087.0683 | -0.9893 | 0.0255 |
| *CCNB3* | 4569.504 | -1.2725 | 0.0247 | 46.6718 | -1.3323 | 0.0447 |

**Table S6.** Differences in differentially expressed genes using DESeq2 for targeted and total RNAseq for sarcomatoid/poorly differentiated carcinoma versus sarcoma.

| *Analysis* | *#* | *Genes identified (adjusted p<.05) in order of significance* |
| --- | --- | --- |
| Identified only by targeted panel | 27 | *MET MYOD1 OMO SNCAIP SOX10 GLl1 GRM1 FGIFR4 CREB3L4 GATA3 MYCN POU5F1 ROS1 MEF2C FOXR2 CREB3L3 NTRK3 MN1 BEND2 NUTM1 VGLL2 PAX8 TTYH1 SERPINE1 SLC45A3 ETV5 COX1* |
| Additional identified by RNAseq | 2539 | *PNCK C9orf152 CLMP FSTL 1 MGC57346-CRHR1 MMP2 OOC-AS1 KCNMA1 PKNOX2 FIBIN LOC80078 TCONS 00029157 KCNMB2-AS1 BTG2 ITGA5 GOLT1A CNTNAP1 BBOX1 C100rf25 XDH PBX4 STOX1 CXCR4 CUBN HYMAI AOAM12* *HSD17B2 SLC52A1 MIR6087 GALNT3 TMEM139 TMC4 ANGPTL5 LINC01279 SLC6A11 STYK1 HMGCS2 CNGB1 EPB41L4B MMP7 MITF WISP2 RTN1 PRKCG NOS2 TBX10 C15orf48 REEP6 FNBP1 L IPAOl1 ZEB1 PON3 OCLK3 FAT4 NW01 CCOC88B PPIP1 R13B ERV3-1 KCNK3 MSRB3 GOA XK CAPNS2 PDE1C RAB31P SEMA40 CEACAM6 MREG FGFR1 ANKS4B SUPT20HL 1 BANK1 HLA-DRB5 MIR4435-2HG IGSF3 UGT1A1 LRRN2 TMEM132E EVC 83GAT1 SORL 1 PCOLCE TMEM184A CXAOR IRAIN SERPINF1 OKK2 LAX1 TP53AIP1 LHFPL2 CCL22 CCOC64 IRX5 FAM129C HS011B2 SLC26A10 LRFN5 S1PR2 HNMT STK26 IPAPSS2 ENTP03 CYP2C19 A2M COL9A2 SLC02B1 SIGLEC9 ANXA3 TLR1 TEK HNF1A-AS1 AP1S2 SLC6A10P CECR2 ALDOC C1orf61 SRRM4 EPHX3 PRR16 OLX2 SLC1A7 SELL LOC727751 LOC729970 LGl2 OOX53 C16orf45 TSPAN13 GFIPT2 LINC00261 CCL 19 SPIRE2 FOXJ1 NKD1 FOXE1 TNFRSF9 RIPK4 KLK8 TMEM63C OUSP4 LAIR1 POON A2ML 1 HS3ST1 MSMB OMTN GRAM02 TM4SF4 LOC101929694 COR02A MAOA LOC283299 APOE RASGRF1 VAT1L BPIFB2 NRARP KJM1211L GJB1 SYT13ADGRF1 INSRR AOGRE4P PRR19 MZB1 FABP5 LAMP3 DNM1 SLC16A5 UGT1A10 LLGL2 PLIN2 ENTP08 SAA2-SM4 TICAM2 KLRG2 MMP16 T FRSF10A FAM159A PA2G41P4 P4HA3 KJM1324 HPCA TRIM29 EMILI 11D1 IPXO MPP4 COS1 PPP1R9A TNXB BIRC7 FAM110C CDH15 VIM-AS1 BCAM CDG TOX3 TORD5 PTRF CRHBP GUCY2C MMP15 􀁬DRA2A NPR1 ASS1 BARX2 SLC9A9 RGS4 EDNRA B3􀁮T5 RAG1 PPFIA3 RHOH MRGPRF HHLA2 CAPN1 LOC654342 AKR1B15 PRSS21 LTBP1 PCDH18 C3orf52 OPYD RGL1 MY05C LOC100507346 NKAIN3 DNAH17-AS1 OXTR SDC2 SLITRK4 CPEB1 POF1B PCDHGA1 IKZF3 QKI ZBTB18 IPALM2 AOGRG5 NACAD OAPP1 TMEM125 MFAP5 CST3 CHRNB2 SEZ6 FCGBP VTCN1 SAPCD2 TFAP2B CLEC3B PKH01 INPP5J MEG8 NEBL IPLCXD2 CLEC4GP1 ITPRIPL2 EPGN ADAMTS2 MYH13 SPRY2 HEPHL 1 OSR2 GSTP1 LOC100505666 SLAIN1 SLC9A3R1 ABCC11 SLC9A3 GALNT18 PCSK6 NMLADL2 ARHGEF38 GPRC5C OCSTAMP COLCA 1 IPALM2-AKAP2 KISS1R KRT15 C10orf99 PERP G'Fl1 FHL 1 RTN4RL 1 HK3 KRT17 SYTL 1 OLEC1 ATP10A RSP02 RBM38 LOXL 1 EML5 IGSF8 SPINK5 PCOHGC5 MEGF10 PRRX1 UG0898H09 FAM83H UNC5CL ARHGEF16 SNORA54 PRNO T JP3 MILR1 OLFML2A KIF19 HID1 COHR1 SOWAHB CSTA CHRM4ATP7B TFEC TMEM25 RAB38 PROX1 PPP1 R 1 B C1orf145 CAPN6 RIC3 PPM1 H LAMB3 PPL ZMIZ1-AS 1 VIT LGALS7B KCNK10 LINC01342 RALGPS1 EFNA2 RASSF4 CCAT1 TMEM238 PLA2G6 HRH2 PRSS22 GJB5 SOX9 CYP3A5 ZFPM2A2M-AS1 PCOHGB4 VWA2 IPIEZ02 SPRiR2F PITX1 VSTM4 PRR36 PAFAH1B3 PDGFRB FHOC1 ZNF107 PACSIN3 MUC5B NOXA1 NORG2 CAMK2B SVEP1 GRIP1 FANCA LOC729737 MUC2 SAMD10 IGSIF9 GRHL 1 SRMSATP884 COL23A1 AN09 EOAR NE004L SLC2A10 VSIG1 TMEM179 IL 1A CPVL PDGFD TRAM2 BEX2 FAM3B PSD2 KCN03 PAWR UPK3B TRIL TMEM79 ASTN2 BVES AKR1C4 PROSER2 NUGGC KCNH4 COL5A2 SIX2 MGC70870 MIR614 SELP RAB9B LRRC56*  *AJP ANXA8 CFAP43 SERPINB13 AOC1 UPK1B EPS8L3 DNAJB4 UGT1A8 THY1 LRBA ASCL2 PCOHGA10 KRT8 SHH UCA1 MAP7 MST1P2 HAND2 TMPRSS4 MARVELD3 TMPRSS13 C1orf115 LRRC16B FLJ13224 AKAP2 TEX9 LVCAT1 LINC00887 RASSF10* |
